# Ensemble Extreme Learning Machine Method for Hemoglobin Estimation Based on PhotoPlethysmoGraphic Signals

**DOI:** 10.3390/s24061736

**Published:** 2024-03-07

**Authors:** Fulai Peng, Ningling Zhang, Cai Chen, Fengxia Wu, Weidong Wang

**Affiliations:** 1 Medical Rehabilitation Research Center, Shandong Institute of Advanced Technology, Chinese Academy of Sciences, Jinan 250100, China; 2 School of Basic Medical Sciences, Shandong University, Jinan 250012, China; 3 Department of Biomedical Engineering, Chinese PLA General Hospital, Beijing 100853, China

**Keywords:** hemoglobin concentration, non-invasive detection, PPG signal processing, extreme learning machine

## Abstract

Non-invasive detection of hemoglobin (Hb) concentration is of great clinical value for health screening and intraoperative blood transfusion. However, the accuracy and stability of non-invasive detection still need to be improved to meet clinical requirement. This paper proposes a non-invasive Hb detection method using ensemble extreme learning machine (EELM) regression based on eight-wavelength PhotoPlethysmoGraphic (PPG) signals. Firstly, a mathematical model for non-invasive Hb detection based on the Beer-Lambert law is established. Secondly, the captured eight-channel PPG signals are denoised and fifty-six feature values are extracted according to the derived mathematical model. Thirdly, a recursive feature elimination (RFE) algorithm is used to select the features that contribute most to the Hb prediction. Finally, a regression model is built by integrating several independent ELM models to improve prediction stability and accuracy. Experiments conducted on 249 clinical data points (199 cases as the training dataset and 50 cases as the test dataset) evaluate the proposed method, achieving a root mean square error (RMSE) of 1.72 g/dL and a Pearson correlation coefficient (PCC) of 0.76 (*p* < 0.01) between predicted and reference values. The results demonstrate that the proposed non-invasive Hb detection method exhibits a strong correlation with traditional invasive methods, suggesting its potential for non-invasive detection of Hb concentration.

## 1. Introduction

Hemoglobin (Hb) is an iron-containing protein in red blood cells that plays an important role in transporting oxygen in the human body. Maintaining an appropriate Hb concentration is vital, as deviations from the normal range may indicate various chronic diseases, including cardiopulmonary disease, kidney disease, tumor-related conditions, and complications during pregnancy [1]. Accurate measurement of blood Hb concentration level is essential for numerous clinical applications, such as anemia screening and blood transfusion guidance [2]. Traditional methods for measuring Hb concentration typically require invasive procedures, in which blood samples are firstly drawn from fingertip or vein by healthcare personnel, and then tested by professionals using inspection equipment [3]. Despite their relatively high accuracy, these invasive methods have inherent limitations. The invasive nature of blood collection poses physical and psychological burdens on patients, increasing the risk of infection. Moreover, traditional invasive approaches lack the ability to monitor changes continuously and dynamically in Hb concentration trends, thereby limiting their capacity to provide timely guidance for intraoperative blood transfusions. In addressing these challenges, non-invasive methods, aim to offer a more patient-friendly and dynamic approach for monitoring Hb concentration, potentially revolutionizing the field by providing real-time insights without the drawbacks associated with invasive techniques.

In recent years, non-invasive detection of Hb concentration has gained great interest. Numerous methods have been proposed, which can be roughly categorized into imaging-based methods and photoplethysmography-based methods. For portability and ease of use, some researchers predict Hb using imaging methods that can be easily captured by the camera or cellphone. Hasan et al. [4] determined the region of interest in the smartphone video and randomly input the extracted features into the artificial neural network (ANN) to estimate Hb concentration. Fan et al. [5] proposed a smartphone-based biosensor to predict Hb concentration using multiple linear regression, showing that the “a” parameter has better performance than the “R” parameter for predicting Hb concentration in RGB color space. Dimauro et al. [6] published a novel public Eyes-defy-anemia dataset and developed a decision-making system based on the RUSBoost classifier to support the automatic diagnosis of anemia. Li et al. [7] developed a snapshot hyperspectral camera using a CMOS chip from the perspective of hyperspectral imaging technology and explored the non-invasive detection of Hb, platelets and total bilirubin in blood components. The imaging methods could predict the hemoglobin concentration conveniently by processing the image captured by the camera; however, the various skin color levels and exposure intensities may have a negative effect on the predicted result. The PPG based methods which can eliminate the effect of skin color differences on pulsatile component were studied by many scholars. Golap et al. [8] extracted PPG signal from fingertip videos utilizing a genetic algorithm for feature selection and established a Hb regression model based on the multi-gene genetic programming (MGGP) algorithm. Tian et al. [9] compared the partial least squares (PLSR) method and the back propagation artificial neural network (BP-ANN) method in predicting Hb concentration and selected samples from the calibration set based on the joint x-y distance (WSPXY) method to improve the model performance. Acharya et al. [10] proposed a multi-model stack regression algorithm including a two-level regression learner to estimate Hb concentration and explored the effect of pregnancy status as a feature on the prediction results, which showed that there is no significant difference in prediction results whether pregnancy status is used or not. Hossain et al. [11] established a two-finger model using digital volumetric pulse waveforms to estimate the percentage of glycated Hb, indicating that the full finger model has high accuracy. Kwon et al. [12] developed a device for collecting PPG signals and utilized ensemble random forest (RF) and extreme gradient boosting to predict Hb concentration. In our previous study [13], we developed a portable non-invasive Hb detection system based on eight channel PPG signals. Partial least squares (PLS) and backpropagation artificial neural network (BP-ANN) algorithms were used to construct the Hb prediction model, and the result showed that the system has the potential for non-invasive Hb detection. Zhu et al. [14] developed a non-invasive hemoglobin detection device based on multi-wavelength photoplethysmography. Several regression models including AdaBoost, BP-ANN and RF were used in their work to construct a hemoglobin prediction model. Results showed that their method can achieve effective non-invasive hemoglobin detection performance. Although these methods have achieved certain results, they have not been widely applicable to clinical devices, and their robustness and accuracy still need to improve.

To further improve the performance of non-invasive Hb detection, in this study, we propose an ensemble extreme learning machine algorithm, named EELM, to estimate Hb values based on PPG signals collected from eight-wavelength LEDs.

## 2. Materials and Methods

**Figure 1:**
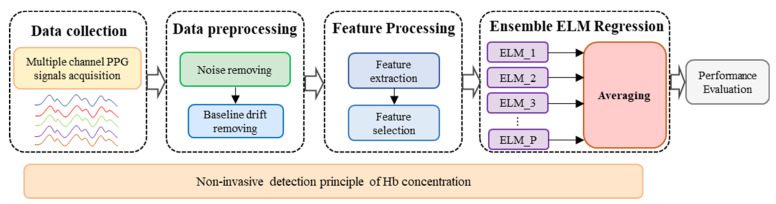
Process of non-invasive Hb detection.

The process of the proposed Hb detection method is shown in Figure 1, which consists of four main procedures: data collection, data preprocessing, feature processing, and ensemble ELM regression. 

Firstly, a non-invasive detection mathematical model of Hb values is derived based on the Beer-Lambert law, which is the theoretical basis of this study. Then, multiple channel PPG signals are captured by a self-designed system. The captured signals are subsequently preprocessed to remove high frequency noise, baseline drift and artifacts. Thirdly, the features that represent the Hb concentration are extracted from the PPG signals according to the derived mathematical model and then simplified by the feature selection process. Finally, an ensemble extreme learning machine (EELM) regression model is established to estimate the Hb concentration.

### 2.1. Non-Invasive Detection Principle of Hb Concentration

The principle of the non-invasive Hb detection method based on PPG signal is the Beer-Lambert law [15], which describes the relationship between the absorbance, the concentration of the substance, the molar extinction coefficient, and the path length of light propagation in the substance. The relationship can be expressed as follows:

(1) $$I=I_{0}e^{-\varepsilon (\lambda )cd}$$

where $I_{0}$ and I describe the intensity of the incident light and the outgoing light, respectively. ε(λ), c and d represent the molar extinction coefficient of the substance at wavelength λ, the concentration of the light-absorbing substance and the path length of light propagating in the medium, respectively.

The absorbance can be obtained from Equation (1) as follows:

(2) $$A=-\ln\frac{I}{I_{0}\;}=\varepsilon (\lambda )cd$$

The skin, muscles, fat, and bones of the human body are fixed tissues, in which the degree of absorption and scattering of incident light are constant for a specific subject. However, the intensity of transmitted light that passes through arterial blood pulsates with the heart beating provides a feasible method to detect Hb concentration by measuring the changes in transmitted light intensity, eliminating the effect of fixed tissues. When the wavelength of incident light is $\lambda_{1}$, the change in absorbance caused by arterial blood pulsation can be expressed as follows:

(3) $$\Delta A_{1}=\left[\varepsilon_{1,1}\left(\lambda_{1}\right)c_{1}+\varepsilon_{1,2}\left(\lambda_{1}\right)c_{2}\right]\Delta <L>$$

where $c_{1}$ and $c_{2}$ are the concentrations of oxygenated Hb and reduced Hb, respectively. Δ<L> represents the change of the average optical path length caused by arterial blood pulsation, $\varepsilon_{1,1}\left(\lambda_{1}\right)$ and $\varepsilon_{1,2}\left(\lambda_{1}\right)$ represent the absorbance of oxygenated Hb and reduced Hb to light with wavelength $\lambda_{1}$, respectively.

Similarly, when the incident light with a wavelength $\lambda_{2}$ illuminates the human tissue:

(4) $$\Delta A_{2}=\left[\varepsilon_{2,1}\left(\lambda_{2}\right)c_{1}+\varepsilon_{2,2}\left(\lambda_{2}\right)c_{2}\right]\Delta <L>$$

where $\varepsilon_{2,1}\left(\lambda_{2}\right)$ and $\varepsilon_{2,2}\left(\lambda_{2}\right)$ are the absorbance of oxygenated Hb and reduced Hb to light with wavelength $\lambda_{2}$, respectively.

The optical path length Δ<L> can be removed by the ratio of Equations (3) and (4).

(5) $$\frac{{\Delta A}_{1}}{{\Delta A}_{2}}=\frac{\left[\varepsilon_{1,1}\left(\lambda_{1}\right)c_{1}+\varepsilon_{1,2}\left(\lambda_{1}\right)c_{2}\right]\Delta <L>}{\left[\varepsilon_{2,1}\left(\lambda_{2}\right)c_{1}+\varepsilon_{2,2}\left(\lambda_{2}\right)c_{2}\right]\Delta <L>}=\frac{{\left[(-\ln\frac{I_{max}}{I_{0}})-(-\ln\frac{I_{min}}{I_{0}\;})\right]}_{\lambda_{1}}}{{\left[(-\ln\frac{I_{max}}{I_{0}\;})-(-\ln\frac{I_{min}}{I_{0}\;})\right]}_{\lambda_{2}}}\approx \frac{{\left(\frac{AC}{DC}\right)}_{\lambda_{1}}}{{\left(\frac{AC}{DC}\right)}_{\lambda_{2}}}$$

In general, the total Hb concentration can be considered as the sum of oxygenated and reduced Hb concentrations. By further theoretical derivation, the total Hb concentration can be obtained as follows:

(6) $$c_{tHb}=f(R_{12},\cdots R_{1N},R_{21},\cdots R_{2N},\cdots R_{N1},\cdots R_{N,N-1})$$

$R_{ij\;}$ in the above formula is defined as follows:

(7) $$R_{ij}=\frac{R_{i}}{R_{j}}=\frac{{AC}_{\lambda_{i}}/{DC}_{\lambda_{i}}}{{AC}_{\lambda_{j}}/{DC}_{\lambda_{j}}}$$

where ${AC}_{\lambda_{i}}$ and ${DC}_{\lambda_{i}}$ are the amplitude strengths of alternating current (*AC*) and direct current (*DC*) of the PPG signal obtained by wavelength of ${\;\lambda }_{i\;}$, respectively.

Based on the above derivation result, it can be seen that the Hb concentration has a certain mapping relationship with the feature information, which can be extracted from the PPG signals.

### 2.2. PPG Signal Acquisition System

A PPG signal acquisition system is implemented based on the mathematical model for non-invasive hemoglobin detection outlined above. This system is designed to capture multiple PPG signals utilizing eight different wavelength LED light sources, namely 610 nm, 630 nm, 660 nm, 690 nm, 750 nm, 805 nm, 850 nm, and 940 nm. The signal measurement position is the finger, which is subjected to sequential irradiation by the eight light sources employing a time multiplexing mechanism.

As light passes through the tissues, it undergoes absorption by hemoglobin in the blood. The extent of absorption is directly correlated with hemoglobin concentration and the absorption coefficient. A PIN photodiode captures the transmitted light, generating an output current proportional to the detected light’s intensity. Subsequently, this current is converted to voltage, amplified, and ultimately transformed into a digital signal through an analog-to-digital converter.

**Figure 2:**
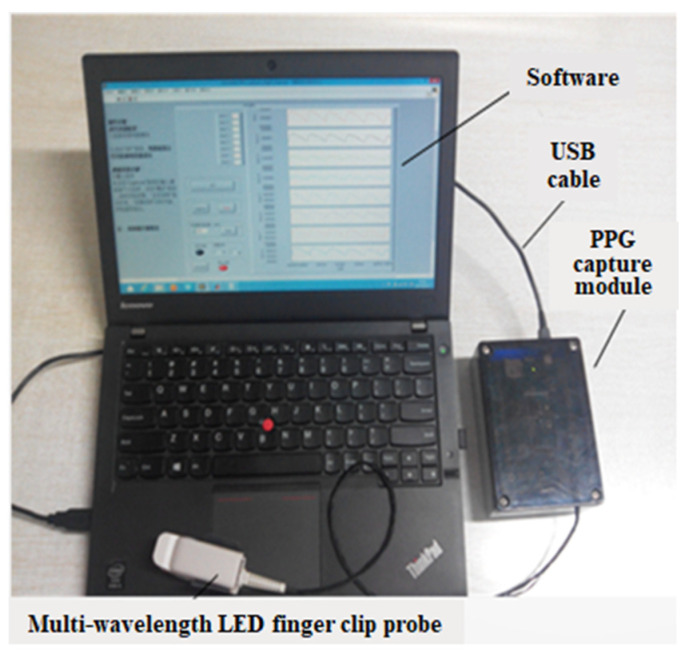
PPG signal acquisition system prototype.

The PPG signal acquisition system prototype is shown in Figure 2, illustrating the integration of these components to facilitate the non-invasive measurement of hemoglobin levels.

### 2.3. Data Preprocessing

Due to adverse factors such as external environmental interference, internal noise of components, and unexpected waveform changes in signal acquisition, the collected PPG signal inevitably contains a large amount of noise interference. In order to ensure the quality of subsequent model construction, it is necessary to preprocess the PPG signal firstly to remove unwanted noise, such as high-frequency noise, power frequency interference, and baseline drift.

Due to the fact that the PPG signal reflects the intensity changes of the outgoing light caused by arterial blood pulsation, its base frequency is usually in the range of 1 Hz~10 Hz. Therefore, this paper first uses a limited impulse response low-pass filter with a cutoff frequency of 10 Hz to filter out high-frequency noise. In addition, during the signal acquisition process, the baseline component of the PPG signal will slowly drift due to breathing and changes in the subject’s posture, which is called baseline drift. The baseline drift, which may have an adverse effect on subsequent feature information, must be filtered out. In this paper, the wavelet transform is used to extract and eliminate baseline drift. Firstly, the input PPG signal is decomposed by a “coif5” wavelet. When the scale is 7 or above, the signal is mainly the baseline drift component. After the PPG signal is decomposed into 7 layers, the approximation coefficient of the 7th layer is set to zero, and finally the remaining layers are reconstructed to obtain the PPG signal without baseline drift. Regarding the PPG signal artifact, particularly the motion artifact, a signal processing method based on a comb filter, as presented in our previous study [16], is employed to mitigate the artifact. The PPG signal period is initially estimated using FFT and spectral peak tracking from the denoised signal. Subsequently, a comb filter is designed based on the obtained PPG signal period to effectively reduce the artifact from the PPG signal.

### 2.4. Feature Extraction and Selection

**Figure 3:**
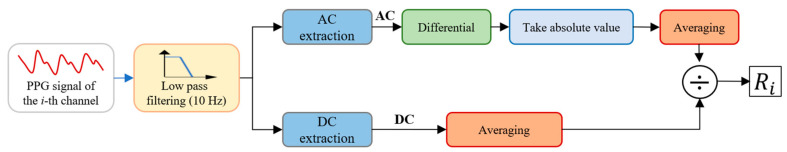
Feature extraction process.

Feature information is extracted from the PPG signals according to the Hb concentration detection model derived above. The preprocessed PPG signals are first windowed with a length of 5 s, and then feature values are calculated according to Formula (7). The AC component is extracted using the differential method, and the average amplitude of the low pass filtered PPG signal is used as the DC component. The PPG signal feature extraction process for each channel is shown in Figure 3. Fifty-six feature values can be obtained from the eight-channel PPG signals. In addition, Hb concentration is related to the age and gender of patients, so the age and gender features are added to the feature information set for Hb concentration prediction.

Since the extracted feature information may involve cross-redundancy, in order to provide concise and accurate feature information for subsequent prediction model construction, the Support Vector Regression-Recursive Feature Elimination (SVR-RFE) algorithm based on the idea of sequential backward selection is used to select the top few features with the highest contribution to regression. The SVR-RFE algorithm can be summarized as follows:

(1) Train the SVR model using all features;
(2) Get the importance of each feature;
(3) Sort features in descending order of importance;
(4) Remove the least important feature to get a new feature set;
(5) Calculate model performance based on the new feature set;
(6) Check whether the feature set is empty? If yes, go to (7); if no, go to (4);
(7) Select the feature set with the highest model performance.

**Figure 4:**
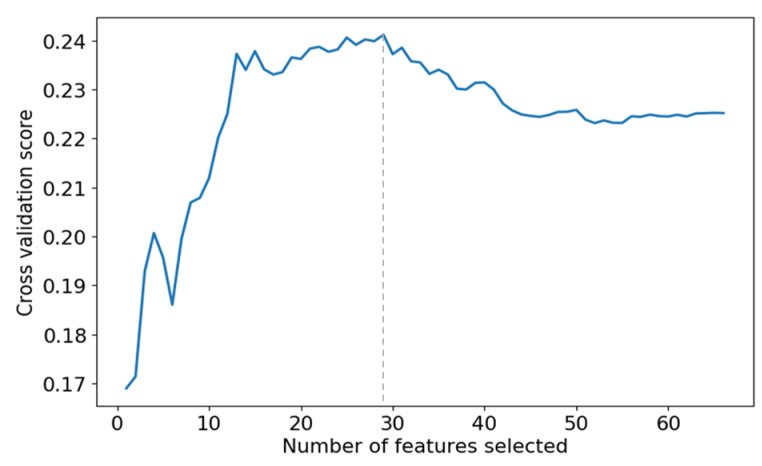
The effect of different numbers of features on the prediction accuracy of SVR models.

The selected results are shown in Figure 4, which shows that the prediction accuracy of the SVR model reaches its best when the top 29 features are selected. Therefore, we select the first 29 features for regression model construction.

### 2.5. Ensemble Extreme Learning Machine

#### 2.5.1. Extreme Learning Machine

**Figure 5:**
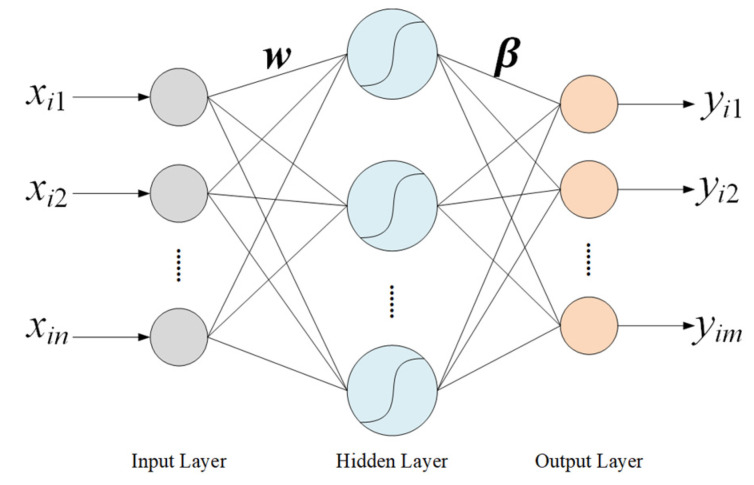
Structure of the ELM algorithm.

Extreme Learning Machine (ELM) [17] is a single hidden layer feedforward neural network algorithm, as shown in Figure 5, that is often used to solve the problems of classification and regression. The weights between the input layer and the hidden layer are randomly initialized and remain unchanged, the weights between the hidden layer and the output layer are calculated during the model training process. The whole process does not require complex iterations and parameter tuning, so ELM has a faster training speed compared with traditional neural network algorithms. In addition, there are only two hyperparameters, the number of nodes in the hidden layer and the activation function.

For the input data $x_{i}={\left[x_{i1},x_{i2},\ldots ,x_{in}\right]}^{T}$, and the predicted output $y_{i}={\left[y_{i1},y_{i2},\ldots ,y_{im}\right]}^{T}$, the relationship between $x_{i}$ and $y_{i}$ is as follows:

(8) $$y_{i}=\sum_{j=1}^{K}\beta_{j}g(w_{j}x_{i}+b_{j}),\;i=1,2,\ldots ,N$$

where $w_{j}$ and $\beta_{j}$ represent the weight vector between input neurons and the jth neuron of the hidden layer and the weight vector between the jth neuron of the hidden layer and the output neurons, respectively. *N* represents the total number of samples, and g(x) represents the activation function. Moreover *n*, *m*, and *K* are the total number of input, output, and hidden layer neurons, respectively.

The above formula can be written in the form of the following matrix.

(9) Hβ = Y

(10) $$H={\left[\begin{array}{c} \begin{array}{c} g(w_{1}x_{1})+b_{1}\cdots \;g(w_{k}x_{1})+b_{k}\\ \vdots \;\cdots \;\vdots \end{array}\\ g(w_{1}x_{N})+b_{1}\cdots \;g(w_{k}x_{N})+b_{k} \end{array}\right]}_{N\times K}$$

(11) $$\beta ={\left[\begin{array}{c} \begin{array}{c} \beta_{1}^{T}\\ \vdots \end{array}\\ \beta_{K}^{T} \end{array}\right]}_{K\times m},Y={\left[\begin{array}{c} \begin{array}{c} y_{1}^{T}\\ \vdots \end{array}\\ y_{N}^{T} \end{array}\right]}_{N\times m}$$

From Equation (9), the expression for β is given as follows:

(12) $$\beta =H^{+}Y$$

where $H^{+}$ is the Moore-Penrose generalized inverse matrix of the matrix *H*.

#### 2.5.2. Average-Based Extreme Learning Machine

The ELM algorithm performs well in regression tasks with relatively short training runtimes [18]. However, the weights from the input layer to the hidden layer are randomly initialized, so different initializations may lead to different regression results, indicating that the training process of ELM is unstable. 

**Figure 6:**
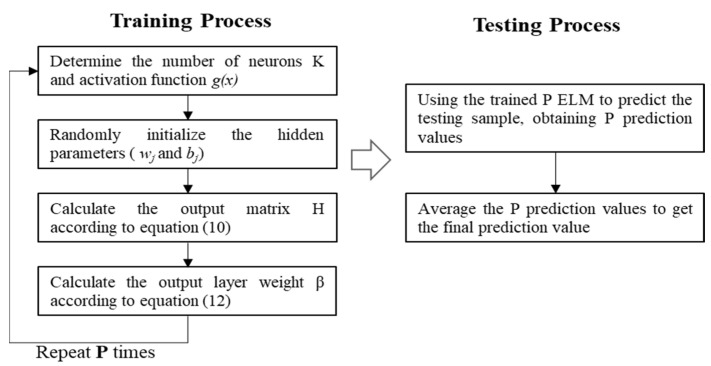
Flow chart of the EELM algorithm.

To address the above problem and improve the stability of ELM, an ensemble ELM algorithm is proposed to predict Hb concentration, which is mainly divided into two steps: establishing multiple independent ELM regression models and integrating the output of each ELM model by the average method to get the final result, as is shown in Figure 6. In fact, the ELM-based learners are different because the hidden parameters of the base ELMs are independently and randomly initialized, guaranteeing the diversity of the basic algorithms in the ensemble learning process. During the training process, P sets of output layer weights are obtained by training P ELM-based learners simultaneously. During the testing process, the test data is fed into the trained ELMs, respectively, to get P prediction results, which are then averaged as the final output.

## 3. Experimental Protocol

### 3.1. Data Collection

249 volunteers were recruited to participate in the experiments with informed consent signed and the process of the experiment was approved by the Institutional Review Board of the Chinese PLA General Hospital. Before the experiments, the volunteers are required to rest for 5 min to maintain emotional stability. During the measurement process, 20 μL of blood was collected from the tip of each subject’s finger, and the hemoglobin concentration was detected by an automatic blood cell analyzer (XS-1000i, Sysmex Corporation, Kobe, Japan), from which the detected Hb value was obtained and used as the reference value. At the same time, the eight-wavelength PPG signal acquisition system was employed to collect signals from the index finger of the hand that did not draw blood for a duration of 2 min with a sample rate of 100 Hz, and the collected data was saved for subsequent processing. The basic physical characteristics of the participants are shown in Table 1.

### 3.2. Experimental Settings

The collected 249 samples are randomly divided into a training set with 199 samples and a test set with 50 samples in an 8:2 ratio. In the experiment, the sigmoid activation function is employed, and the number of base ELM units in EELM is set to 200, with each ELM having 20 hidden layer nodes. The model is trained 200 times, with the data set randomly divided each time, and the average value is taken as the result of the evaluation metric. 

### 3.3. Performance Metrics 

The method proposed in this paper is evaluated from three aspects: statistical analysis (Root mean square deviation (RMSE) and Pearson correlation coefficient (PCC)), consistency analysis (Bland-Altman plots), and Error grid analysis.

(a) Statistical metrics

The RMSE evaluates the similarity of two samples from the perspective of error, but cannot measure correlation. Therefore, the PCC is used as a supplementary evaluation indicator to calculate the correlation between the predicted and reference values.

(b) Bland-Altman Plots

The Bland Altman plot [19] is used to estimate the consistency between two Hb measurement methods (invasive and non-invasive). Generally speaking, the method is considered reliable when most of the observed differences are within the 95% consensus range (±1.96 standard deviation).

(c) Error grid analysis

The error grid analysis describes the difference between the actual and estimated Hb values by a plot, which is segmented into several regions depending on the error level. The Clarke error grid analysis (CEGA), a gold criterion for evaluating the clinical accuracy of blood glucose monitors, was modified to assess the Hb estimators by many studies [10,20], which is also applied in this study. In the current study, the plot is also segmented into three regions (i.e., A, B and C) based on the Error grid. 

Region A encompasses values that deviate from the actual values by no more than ±1 g/dL. Subjects within this region can ensure the correct clinical treatment. Values outside of Region A, with an error within ±2 g/dL, are allocated to Region B. Subjects in Region B will not receive inappropriate treatment. Values that fall outside the scope of Region A and Region B, with an error exceeding ±2 g/dL, are assigned to Region C. Subjects in Region C may be at risk of inappropriate clinical treatment.

## 4. Results

**Figure 7:**
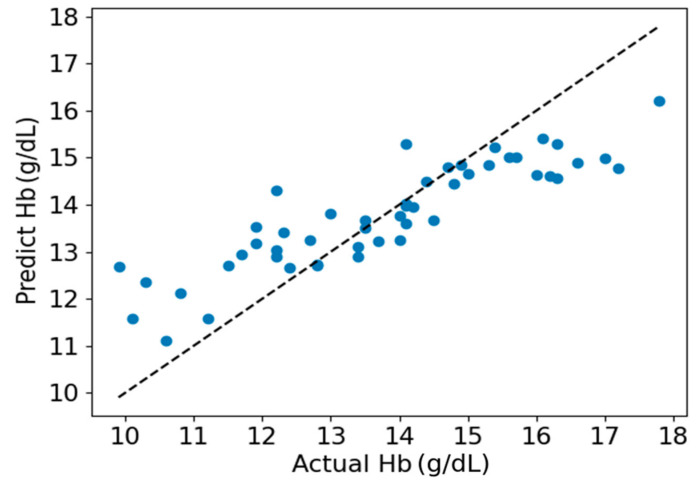
Scatter plot of estimated and actual Hb values.

**Figure 8:**
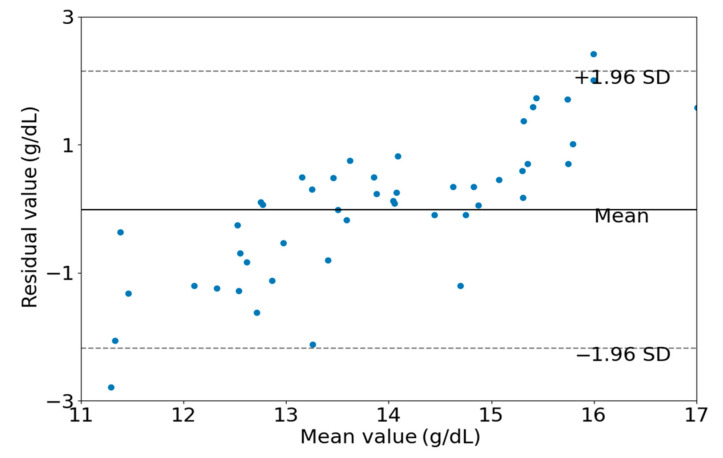
Bland-Altman descriptive plots of estimated and actual values.

As shown in Figure 7, a scatter plot between the estimated Hb values obtained by the EELM method and the reference Hb values is presented. Figure 8 shows the Bland-Altman descriptive plot between the reference and predicted values of Hb, with the horizontal coordinate being the value of Hb and the vertical coordinate being the residual difference between the predicted value and the reference value. It can be seen that most of the test data for the 50 cases are within the 95% consistency range, indicating a good agreement between the two methods, which suggests that the method designed in this paper is valid for Hb prediction.

To further validate the performance of the algorithm presented in this paper, a comparative analysis was conducted, comparing the proposed EELM against four established regression methods. The selected control methods include Linear Regression (LR), Support Vector Regression (SVR), RF and the traditional ELM.

**Figure 9:**
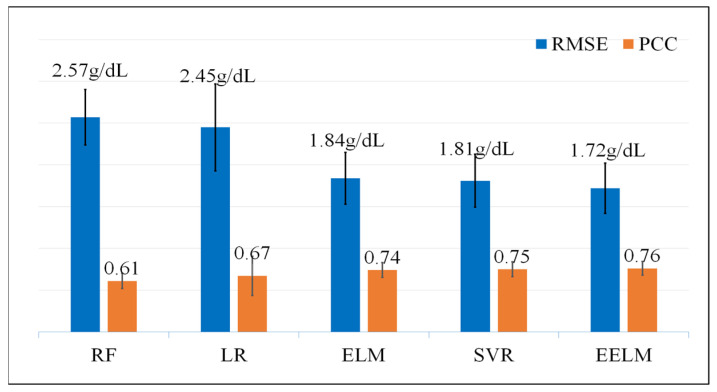
RMSE and PCC values of the five different regression methods, the unit of RMSE is g/dL and the PCC is dimensionless.

Figure 9 shows the RMSE and PCC values of the five different algorithms. The EELM model outperforms the other four models in both evaluation indicators, getting the best performance RMSE of 1.72 g/dL and PCC of 0.76 (*p* < 0.01).

**Figure 10:**
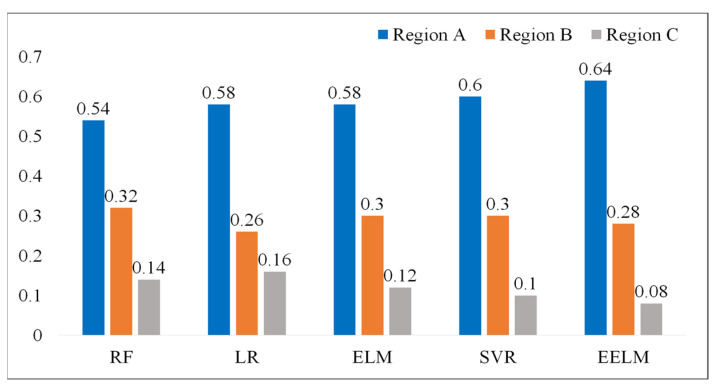
Error grid analysis of the regression models, the values presented in the figure represent the percentage of subjects in different regions.

The result of the error grid analysis for all the five methods is depicted in Figure 10. The EELM model performs the best with the highest percentage of subjects in Region A (64%) and the lowest percentage of subjects in Region C (0.08%).

The computational analysis was also investigated for the real-time applicability of the proposed method by testing the algorithm’s run time in predicting hemoglobin levels. The experiments were run on a laptop equipped with an Intel Core i7-8565U CPU (Intel, Santa Clara, CA, USA) at 1.80 GHz and 8 GB of RAM. The experimental results are shown in Table 2. The run time of all the methods for predicting hemoglobin is deemed acceptable for real-time application scenarios, even though the proposed EELM method exhibits the highest computational load. 

## 5. Discussion

The non-invasive, immediate, and accurate detection of Hb levels possesses important clinical significance for anemia screening and blood transfusion guidance. The non-invasive Hb detection method based on PPG signals is the mainstream approach, which can eliminate the effect of fixed tissue, such as skin, bone and muscle using the PPG alternating component caused by pulsatile blood. The theoretical basis of the PPG signal-based method is the Beer-Lambert law, which describes the relationship between the absorbance of light by a substance and the substance concentration level. In this paper, a non-invasive Hb detection method based on multiple-channel PPG signals is presented. A non-invasive Hb detection model was first derived based on the Beer-Lambert law, then multiple-channel PPG signals were captured using eight-wavelength LEDs with wavelengths varying from 610 nm to 940 nm. In addition to the age and gender features, fifty-six features were extracted from the eight channel PPG signals based on the derived Hb detection model. The features extracted from each channel PPG signal are the ratio between the alternating current and direct current of the same channel PPG signal, which can eliminate the variational influence of emission intensity. Due to the fact that the extracted features inevitably have redundant information, which will have a negative effect on Hb prediction, the SVR-RFE algorithm was used to select out the features that mostly contribute to Hb detection. 

The regression model was established based on the ELM algorithm, which is a single hidden layer feedforward neural network algorithm. The ELM algorithm has excellent nonlinear fitting performance in classification and regression tasks, and it does not require complex iterations and parameter tuning, so ELM has a faster training speed compared with traditional neural network algorithms. However, the weight parameters are randomly initialized, which may lead to instability in the prediction results. To address this problem and improve the stability of the model, we established multiple independent ELM regression models and then integrated the results obtained by each ELM. 

The performance of the proposed method was visually presented in Figure 7 and Figure 8. It is apparent from these figures that hemoglobin values within the range of 13 g/dL to 15 g/dL demonstrate relatively high prediction accuracy, whereas values outside this range exhibit lower prediction accuracy. This observation can be attributed to the abundance of samples with hemoglobin values falling within the 13 g/dL to 15 g/dL range. A sufficient sample quantity ensures diversity in features extracted from PPG signals, thereby contributing to the construction of a robust model for hemoglobin prediction. Conversely, the inadequate number of samples with hemoglobin values outside the range of 13 g/dL to 15 g/dL results in a lack of diversity in feature sets, compromising the robustness of the constructed model for predicting hemoglobin values.

To further assess the effectiveness of the proposed method in comparison to alternative approaches, a comprehensive evaluation was conducted against four established methods, namely LR [21,22], RF [14,23], SVR [24], and ELM. LR was included as a baseline method, given its common use in various regression tasks including hemoglobin detection. RF is a prevalent hemoglobin regression algorithm considered for comparison, given its robustness and versatility. SVR was chosen due to its widespread application and efficacy in hemoglobin prediction scenarios. Additionally, the traditional ELM was included as a control method, serving as a reference point since the proposed EELM is an ensemble version of the ELM. Three common performance metrics, including statistical analysis (RMSE and PCC), consistency analysis (Bland-Altman plots), and error grid analysis, were employed to assess the performance of the proposed method in predicting hemoglobin. The results indicate that the proposed method surpasses the other four methods, achieving the best performance with an RMSE of 1.72 g/dL and a PCC of 0.76 (*p* < 0.01). Additionally, it attained the highest percentage of subjects in Region A (64%) and the lowest percentage of subjects in Region C (0.08%) in terms of error grid analysis. The computational complexity was also examined to assess real-time applicability by measuring the algorithm’s runtime for predicting hemoglobin levels. The experimental results indicate that the proposed method has the longest run time compared to the other four methods, achieving a run time of 26.6 ms for predicting 50 hemoglobin values on a laptop. This longer run time is attributed to the fact that EELM involves the integration of many independent ELMs, leading to a substantial increase in processing time. Despite the higher computational load, the run time for predicting hemoglobin is considered acceptable for real-time application scenarios.

The proposed method is primarily comprised of two stages: the model construction stage and the real-time hemoglobin prediction stage. As indicated by the computational analysis in the results section, the computational load of the proposed method for predicting hemoglobin is deemed acceptable for real-time application scenarios. The prediction stage is adaptable for transfer to other devices, including consumer or research devices, provided that these devices can capture PPG signals using the same wavelength light sources as designed. However, it is worth noting that the model construction process is most efficiently carried out on research equipment, given its relatively high computational requirements. 

Although the proposed method could achieve relatively high prediction performance, there are still some limitations in this study. Firstly, the sample volume is insufficient to robustly establish the model’s generalization ability. Secondly, the Hb values in the samples predominantly fall within the normal range (12~16 g/dL), with a scarcity of samples with abnormal levels, resulting in a relatively high prediction error for the abnormal range.

In future investigations, we aim to overcome the current limitations by significantly augmenting the experimental sample size. Our primary emphasis will be on incorporating a more diverse range of samples, with particular attention given to those exhibiting abnormal Hb values. Our commitment to comprehensive future studies is geared towards refining the system’s performance, with a specific focus on aligning it with the stringent requirements of clinical applications. Moreover, we will rigorously validate the reliability of our detection method beyond laboratory settings. This validation process will extend to non-laboratory environments, ensuring the adaptability and efficacy of our approach in diverse scenarios. This includes the exploration of practical applications in wearable consumer scenarios, thereby expanding the versatility of our detection system. Our goal is to enhance the method’s robustness and applicability, establishing it as a reliable tool for a broader spectrum of real-world situations.

## 6. Conclusions

In this paper, a non-invasive Hb detection method using the EELM algorithm based on eight-wavelength PPG signals is proposed. Firstly, a non-invasive detection model is derived based on the Beer-Lambert law. Secondly, preprocessing techniques such as low-pass filtering, and wavelet transformation are applied to multi-channel PPG signals to filter out high-frequency noise and baseline drift. In addition, the SVR-RFE algorithm is adopted to select the extracted features to improve the accuracy and reliability of Hb concentration detection. Finally, the EELM algorithm is proposed to establish Hb concentration detection model, using 199 samples for training and 50 samples for testing. The RMSE and PCC between the predicted and reference Hb values are 1.72 g/dL and 0.76 (*p* < 0.01), respectively. Compared with the other four common methods, the algorithm proposed in this paper exhibits significant advantages. The experimental results show that the non-invasive detection model of Hb designed in this paper has strong consistency with the traditional invasive detection methods, providing a new option for clinical applications such as anemia screening and dynamic monitoring of intraoperative blood transfusion. In our future work, we plan to expand the experimental sample size and conduct more comprehensive studies to enhance the system’s performance before it meets the requirements for clinical applications.

## Figures and Tables

**Table 1:** Physical statistics of subjects.

Number of Volunteers (Male/Female)	Ages (Year)	Height (cm)	Weight (kg)	SpO_2_ (%)
100/149	41.7 ± 24.3	167 ± 12	67 ± 15	97 ± 2

**Table 2:** Run time of each method in predicting hemoglobin levels.

Methods	RF (N = 50)	LR (N = 50)	ELM (N = 50)	SVR (N = 50)	EELM (N = 50)
Run time	10.28 ms	0.15 ms	0.33 ms	1.63 ms	26.6 ms

## Data Availability

The data are not publicly available due to internal company board policy.
